# Supplementary material for: Non-Additive and Asymmetric Allelic Expression of p38 mapk in Hybrid Tilapia (Oreochromis niloticus ♀ × O. aureus ♂)
Source: Animals (Basel). 2024 Jan 15;14(2):266. doi: 10.3390/ani14020266 (PMC10812652; doi:10.3390/ani14020266)
Supplement: Supplementary file 1 [file animals-14-00266-s001.zip › animals-2717353-supplementary.pdf]

Number	AR(A)	NL(G)
1	25.28	74.72
2	10.21	89.79
3	29.41	70.59
4	26.12	73.88
5	29.63	70.37
6	29.18	70.82
7	3.09	96.91
8	30.27	69.73
9	24.00	76.00
10	28.48	71.52
11	8.57	91.43
12	33.18	66.82
13	11.72	88.28
14	21.39	78.61
15	29.32	70.68
16	27.18	72.82
17	31.25	68.75
18	31.89	68.11
19	24.34	75.66
20	6.59	93.41

Above are the pyrophosphate sequencing results of *p38* gene in the parental species.

Table S1. Pyrophosphate sequencing *p38* expression bias raw data statistics.
